# SAP domain forms a flexible part of DNA aperture in Ku70/80

**DOI:** 10.1111/febs.15732

**Published:** 2021-02-16

**Authors:** Aleš Hnízda, Petr Tesina, Thanh‐Binh Nguyen, Zdeněk Kukačka, Lukas Kater, Amanda K. Chaplin, Roland Beckmann, David B. Ascher, Petr Novák, Tom L. Blundell

**Affiliations:** ^1^ Department of Biochemistry University of Cambridge Cambridge UK; ^2^ Gene Center and Department of Biochemistry University of Munich Germany; ^3^ Computational and Systems Biology Bio21 Institute University of Melbourne Parkville VIC Australia; ^4^ Computational Biology and Clinical Informatics Baker Heart and Diabetes Institute Melbourne VIC Australia; ^5^ Institute of Microbiology Academy of Sciences of the Czech Republic Prague Czech Republic; ^6^ Department of Biochemistry and Molecular Biology University of Melbourne Parkville VIC Australia

**Keywords:** DNA double‐strand break, integrative structural biology, Ku70/80, nonhomologous end joining, SAP domain

## Abstract

Nonhomologous end joining (NHEJ) is a DNA repair mechanism that religates double‐strand DNA breaks to maintain genomic integrity during the entire cell cycle. The Ku70/80 complex recognizes DNA breaks and serves as an essential hub for recruitment of NHEJ components. Here, we describe intramolecular interactions of the Ku70 C‐terminal domain, known as the SAP domain. Using single‐particle cryo‐electron microscopy, mass spectrometric analysis of intermolecular cross‐linking and molecular modelling simulations, we captured variable positions of the SAP domain depending on DNA binding. The first position was localized at the DNA aperture in the Ku70/80 apo form but was not observed in the DNA‐bound state. The second position, which was observed in both apo and DNA‐bound states, was found below the DNA aperture, close to the helical arm of Ku70. The localization of the SAP domain in the DNA aperture suggests a function as a flexible entry gate for broken DNA.

**Databases:**

EM maps have been deposited in EMDB (EMD‐11933). Coordinates have been deposited in Protein Data Bank (PDB 7AXZ). Other data are available from corresponding authors upon a request.

## Graphical abstract

Ku70/80 mediates a recognition of DNA breaks to initiate nonhomologous end joining, an important DNA repair mechanism for maintaining genomic integrity. Our study describes dynamic movements of the SAP domain, a C‐terminal domain in the Ku70 subunit, depending on DNA binding. Using cryo‐EM, mass spectrometry and computational modelling, the SAP domain was localized at the DNA aperture and probably forms a flexible entry gate for broken DNA.

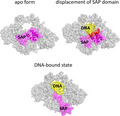

**Abbreviations**
CTD: C‐terminal domain
DSA: Di(N‐succinimidyl) adipate
NHEJ: nonhomologous end joining
TF: transcription factor

## Introduction

DNA double‐strand breaks represent the most dangerous type of DNA lesions that, if not properly repaired, may compromise genomic integrity of cells. Nonhomologous end joining (NHEJ) is an essential repair mechanism that facilitates religation of DNA breaks throughout the entire cell cycle.

This process is mediated by the Ku70/80 complex, which recognizes the ends of the broken DNA and serves as an interaction hub for recruitment of downstream components of the NHEJ pathway [1]. In addition to NHEJ, Ku70/80 also plays an important role in multiple biological processes including telomere maintenance [2], HIV replication [3] and suppression of apoptosis [4].

**Fig. 1:**
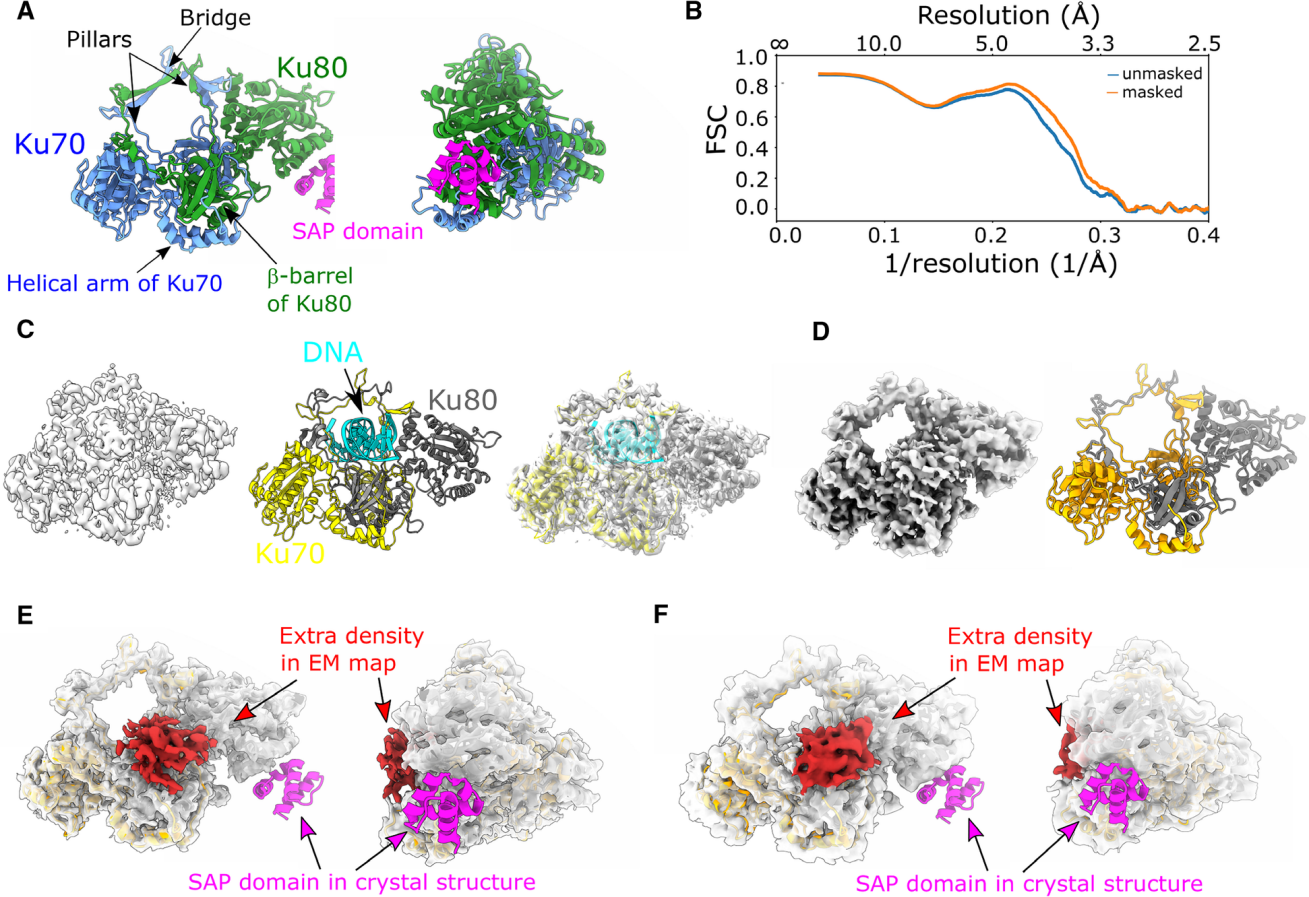
Comparison of the Ku70/80 apo form (Ku70/trKu80) crystal and cryo‐EM structures. (A) The crystal structure of Ku70/80 (PDB ID 1JEQ; front and side views shown). (B) FSC curve of cross‐linked the Ku70/80 apo form. (C) Cryo‐EM analysis of the cross‐linked Ku‐DNA complex revealed no density for the SAP domain. EM density map (left) with its structural model (middle) and their superposition (right) are shown. (D) EM density map (left) and refined structural model (right) for the Ku70/80 apo form cross‐linked with DSA. (E) Superposition of EM density map for the cross‐linked Ku70/80 apo form with its structural model (front and side views shown). The additional density is coloured in red. The position of the SAP domain in the crystal structure is highlighted as a magenta cartoon. (F) Superposition of the EM map for the Ku70/80 apo form lacking cross‐links with its structural model. Colouring is the same as for panel C. Structural images were generated by chimerax.

Ku70 and Ku80 form a pseudosymmetrical heterodimer with a preformed ring (also called DNA aperture) responsible for sequence‐independent DNA binding. The two subunits share a common domain topology, comprising the N‐terminal α/ß domain, the central region (ß‐barrel and bridge connected via pillars) followed by the helical arm (Fig. 1A) [5]. Ku70 contains a C‐terminal domain (CTD), known as the SAP domain, comprising three alpha helices (5 kDa) [6], while Ku80 contains a 19 kDa globular region with superhelical topology [7, 8]. CTDs of both subunits are connected with their respective protein cores via flexible linkers, significantly increasing the conformational flexibility of Ku70/80.

Although the Ku80 CTD is known to be responsible for the recruitment of DNA‐PKcs (DNA‐dependent protein kinase, catalytic subunit) to broken ends [9, 10], the biological function of the Ku70 SAP domain is still enigmatic. The SAP domain comprises three alpha helices with a topology typical for DNA‐binding proteins [5]. Indeed, it binds to DNA but with much lower affinity than the Ku70/80 DNA aperture [6]. Crystallographic study of Ku70/80 has shown that the SAP domain is localized close to the Ku80 α/ß domain but distal to the central DNA aperture. Upon binding of DNA to Ku70/80, the SAP domain no longer binds at this position and probably fluctuates between several states resulting in missing density in the crystal structure of the DNA‐bound state [5]. These structural rearrangements of the SAP domain upon DNA binding have also been observed using low‐resolution cryo‐EM [11] and through limited proteolysis and chemical labelling of the protein surface [12].

Interestingly, the SAP domain was found to be essential for interaction of Ku70/80 with several transcription factors (TFs) containing homeodomains including homeobox C4 (HOX C4) and octamer‐binding transcription factor 1 (OCT‐1) [13]. Intermolecular interaction with DNA‐binding domains from various TFs to Ku70/80 has distinct effects on NHEJ efficiency. While homeobox B7 (HOX B7) enhanced NHEJ efficiency in epithelial cells [14], caudal type homeobox 2 (CDX2) and thymocyte selection‐associated high‐mobility group box protein (TOX) inhibited the repair of broken ends in colon cancer [15] and leukaemia [16]. However, structural determinants for interaction between Ku70/80 and TFs mediated by the SAP domain are largely undefined.

Here, our objective is to characterize interactions of the SAP domain within the Ku70/80 heterodimer. By combining cryo‐EM, mass spectrometric analysis of intermolecular cross‐linking and molecular modelling, we capture the variable positions of the SAP domain and its interdomain interactions within Ku70/80. We also describe the structural rearrangements induced by DNA binding. These insights provide useful information about possible biological functions of the SAP domain.

## Results

### Cryo‐electron microscopy revealed an additional density at the DNA aperture

In order to capture the position of the SAP domain in the Ku70/80 heterodimer, we used intermolecular cross‐linking followed by single‐particle cryo‐EM. We analysed the apo form and the DNA‐bound state treated with Di(N‐succinimidyl) adipate (DSA), an amine‐reactive cross‐linker with a spacer arm length of 8.6 Å [17]. In the case of the apo form, the full‐length Ku70/80 exhibited a high propensity to form aggregates on EM grids, whereas truncated protein Ku70/tr80 (Ku70/80 complex composed of full‐length Ku70 and truncated Ku80 lacking residues 566–732) led to uniform distribution of particles. Therefore, Ku70/tr80 was used for subsequent analysis using cryo‐EM and mass spectrometry.

Cryo‐EM data sets for both the apo form and the DNA‐bound form of Ku70/tr80 were collected to provide enough particles for 3‐D reconstruction (226 000 and 216 000 for apo form and DNA‐bound, respectively). As a result, the structure of the apo form was reconstructed to an overall resolution of 3.2 Å and the DNA bound form to 3.8 Å (Fig. 1B). The structural models were refined according to the obtained EM maps and compared with their crystal structures. In the case of Ku70/80 bound to DNA, the calculated EM density fitted well to the previously described crystal structure (PDB ID 1JEY) in which density for the SAP domain has not been observed (Fig. 1C). The superposition of the crystal and EM structures revealed RMSD for 1021 C_α_ atoms of ≈ 1.2 Å, illustrating only subtle changes between the two models for Ku70/80 bound to DNA.

Next, we compared our EM density map for cross‐linked apo form Ku70/80 with the available crystal structure (PDB ID 1JEQ). This analysis revealed different positions for the SAP domain in the cryo‐EM structure compared with that of the crystal structure (Fig. 1A,D,E), although the dimeric core of the complex did not exhibit any significant changes (RMSD for 1017 C_α_ atoms of ≈ 0.8 Å). In the crystal structure, the SAP domain is localized in close proximity to α‐helices 5 and 6 (aa 145–157 and 200–214) from the α/ß domain of Ku80. These interdomain contacts are mediated via the first helix‐turn region of the SAP domain (aa 559–577) but the presence of only few direct contacts (specifically, three polar interactions and one van der Waals contact) indicate rather weak interaction between the two respective regions. In contrast, the EM map did not contain any density for the SAP domain in this region. Interestingly, an additional density was observed at the dimerization interface close to the DNA aperture (Fig. 1E). This density, however, was apparent only at a lower contour level and was rather fragmented, which did not allow confident model fitting. The position of this additional density was found to be close to a loop which connects the helical arm and the SAP domain in Ku70 (aa 530–537). Moreover, this density fits to an overall size and shape of the SAP domain. These observations indicate that the SAP domain could be localized at the DNA aperture.

To assess the effect of cross‐linking on plausible position of the SAP domain in the EM density map, we analysed the unmodified apo form of Ku70/tr80. We obtained 94 000 particles that allowed us to calculate an EM density map with a resolution of 4.3 Å. Consistent with results obtained for the cross‐linked complex, we observed the same additional density at the DNA aperture (Fig. 1F), which was badly resolved due to high conformational flexibility. This shows that the position of this density was not forced by cross‐linking.

In summary, cryo‐EM analysis of Ku70/80 showed that the SAP domain is not stably bound to the Ku70/80 complex but likely localized at the DNA aperture from which it is displaced upon DNA binding.

### Mass spectrometry captured fluctuating positions of the SAP domain

To further validate the observed position of the SAP domain, we performed mass spectrometric analysis to localize distance constraints in the Ku70/80. In order to do so, we applied the DSA cross‐linker, which was used for our cryo‐EM analysis. To assess conformational changes upon DNA binding, we compared the relative abundance of cross‐links in apo‐ and DNA‐bound states by quantitative experiment using isotopically labelled DSA (^12^C/^13^C) as described previously [18].

**Fig. 2:**
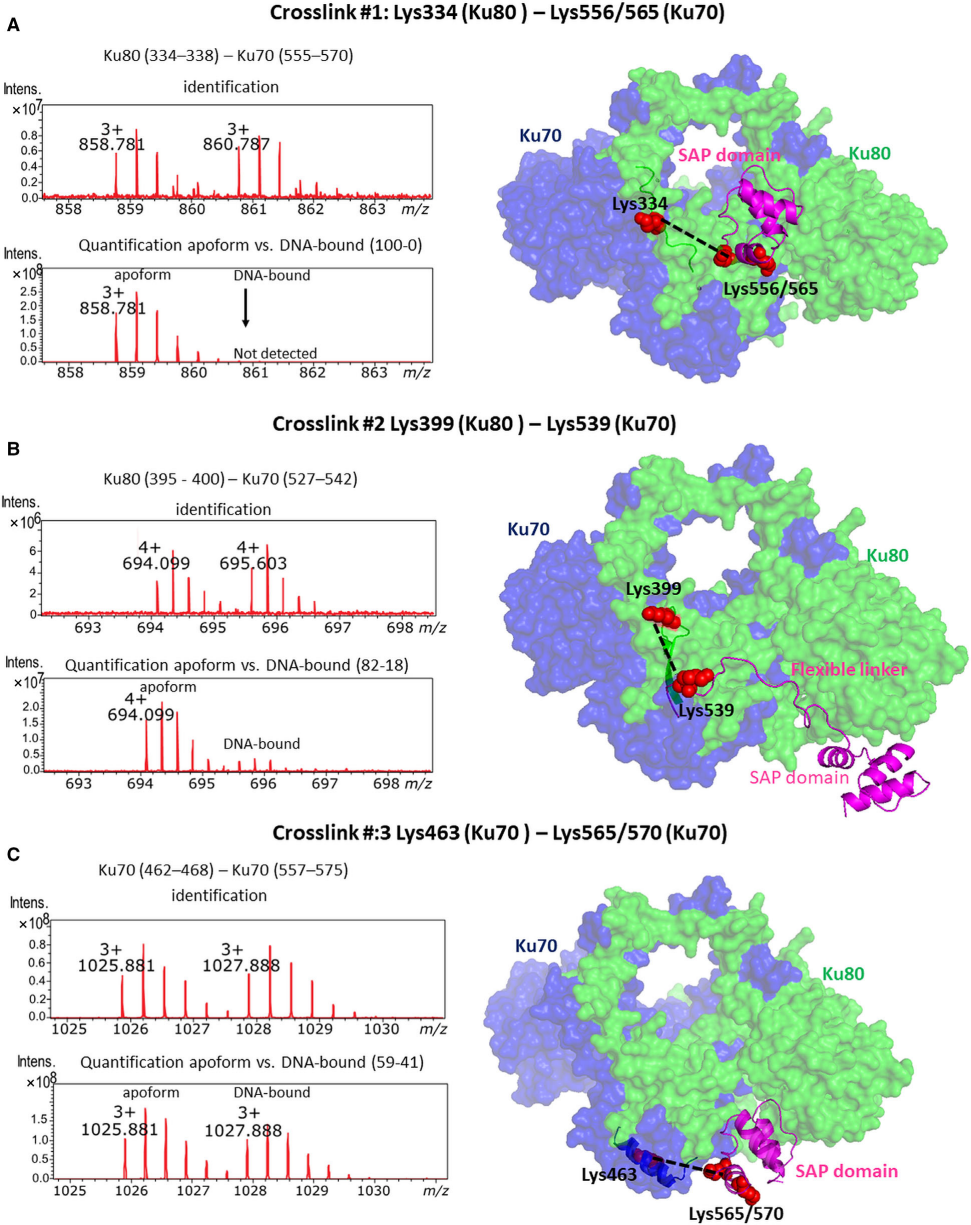
Interdomain cross‐links of lysine residues in the SAP domain. (A) Cross‐link between Lys334 (Ku80) and Lys556/565 (SAP domain in Ku70). (B) Cross‐link between Lys399 (Ku80) and Lys539 (SAP domain in Ku70). (C) Cross‐link between Lys463 (helical arm in Ku70) and Lys565/570 (SAP domain in Ku70). Each panel shows mass spectra for an individual cross‐link alongside the structural model of Ku70/80 including the SAP domain and its flexible linker. Left Mass spectra for identification of cross‐links and quantitative comparison of apo form with DNA‐bound state are shown. Mass spectra for identification (upper part) contained the pair of peaks resulting from isotopically labelled DSA cross‐linker (C12/C13). Quantitative cross‐linking (lower part) shows relative differences between apo form and DNA‐bound state. Right Structural model of the core for the Ku70/80 apo form is based on our EM data and shown in surface representation. Different positions of the SAP domain (NMR model PDB ID 1JJR) including flexible linker are shown only for clear illustration of cross‐linked residues which are highlighted as red spheres. Structural images were generated by pymol.

Using liquid chromatography coupled with Fourier transform ion cyclotron resonance mass spectrometry, we identified 24 cross‐links in the Ku70/tr80 (see Table 1 for a list of cross‐links). The comparison of the mass spectrometric analysis with the available structural models revealed that 14 links were captured within the core of Ku70/80 (aa 35–537 in Ku70 and 5–542 in Ku80), seven links were localized in the SAP domain (aa 538–609) and three links were localized in flexible loops missing in the available structural models (aa 1–34 in Ku70 and 543–565 in Ku80). Further analysis of the cross‐links in the complex core showed that 12 of the 14 (86%) links connected two lysine residues located close to each other in the structural model of Ku70/80. This demonstrated that intermolecular cross‐linking coupled with mass spectrometric analysis of Ku70/80 provided structurally relevant information. The most important, seven cross‐links were found in the SAP domain providing information about this region. While four of these cross‐links represented connections of residues within the SAP domain (intradomain cross‐linking), the other three cross‐links revealed contacts of the SAP domain with different parts of the Ku70/80 complex (interdomain cross‐linking). Specifically, we identified spatial constraints of the SAP domain with the helical arm of Ku70 (aa 450–538) and with the central domain of Ku80 (aa 242–433) forming the DNA aperture (Fig. 2).

**Table 1:** List of cross‐links in Ku70/80 identified by mass spectrometry.

Cross‐link	Localization in the Ku70/80 complex
N terminus (Ku70) ‐ Lys 260 (Ku70)	N‐terminal loop with ß‐barrel
N terminus (Ku70) ‐ Lys 543 (Ku80)	Flexible terminal loops
N terminus (Ku70) ‐ Lys 544 (Ku80)	Flexible terminal loops
Lys 9 (Ku70) ‐ Lys 253 (Ku70)	N‐terminal loop with a linker between ß‐barrel and α/ß domain
Lys 31 (Ku70) ‐ Lys 253(Ku70)	N‐terminal loop with a linker between ß‐barrel and α/ß domain
Lys 31 (Ku70) ‐ Lys 297 (Ku70)	N‐terminal loop with bridge region
Lys 189 (Ku70) ‐ Lys 445 (Ku70)	α/ß domain with a linker between ß‐barrel and helical arm
Lys 189 (Ku70) ‐ Lys 565 (Ku80)	α/ß domain with C‐terminal loop (Ku80)
Lys 260 (Ku70) ‐ Lys 543 (Ku80)	ß‐barrel (Ku70) with pillar region (Ku80)
Lys 282/7 (Lys70) ‐ Lys307 (Ku80)	Pillar region (Ku70) with bridge region (Ku80)
Lys 317 (Ku70) ‐ Lys 282 (Ku80)	Bridge region
K463 (Ku70)‐Lys565/570 (Ku70)	Helical arm with the SAP domain
Lys 468 (Lys70) ‐ Lys 510 (Ku70)	Helical arm
Lys468 (Ku70) ‐ Lys516 (Ku70)	Helical arm
Lys539 (Ku70) ‐ Lys542 (Ku70)	Linker between the helical arm and the SAP domain
Lys539 (Ku70) ‐Lys544 (Ku70)	Linker between the helical arm and the SAP domain
Lys539 (Ku70) ‐ K399 (Ku80)	ß‐barrel (Ku80) with linker between the helical arm and the SAP domain
Lys565/570 (Ku70) ‐ K334 (Ku80)	Pillar region (Ku80) with the SAP domain
Lys575 (Ku70)‐Lys596 (Ku70)	SAP domain
Lys596 (Ku70) ‐ Lys605 (Ku70)	SAP domain
Lys51 (Ku80) ‐ Lys 273 (Ku80)	Pillar region with α/ß domain
Lys273 (Lys80) ‐ Lys399 (Ku80)	Pillar region with ß‐barrel
Lys 291 (Ku80) ‐ Lys 413 (Ku80)	Bridge with ß‐barrel
Lys 532 (Ku80) ‐ Lys 565 (Ku80)	Flexible terminal loop

Cross‐linking between the SAP domain and the central domain of Ku80 was observed in two regions. The first connected Lys 556/565 from the first helix of the SAP domain with Lys 334 localized at a loop connecting the ß‐barrel with the bridge region of Ku80 (Fig. 2A; cross‐link #1). This cross‐linking was detected only in the apo form as DNA binding entirely prevented the linking reaction. This observation further confirms that the contact between the SAP domain and DNA aperture is disrupted by DNA binding. The second cross‐link connected Lys 539 (Ku70) with Lys 399 (Ku80). The Lys 539 residue is localized in a flexible loop of Ku70 (aa 535–558) connecting the helical arm with the SAP domain. In Ku80, Lys399 lies in a loop between two ß‐strands of the ß‐barrel domain, which forms a part of the DNA aperture (Fig. 2B). Connection of the respective regions was predominantly observed in the apo form rather than in the DNA‐bound state. This observation showed a transient contact of the linker connecting the Ku70/80 core with the SAP domain and its displacement due to structural rearrangements upon DNA binding.

The cross‐linking of Lys 463 of the helical arm with Lys 565/570 from the SAP domain in Ku70 demonstrated the spatial proximity of the first helix of the SAP domain (aa 559–577) with α‐helix 14 (aa 455–469) of the helical arm localized under the DNA aperture (Fig. 2C; cross‐link #3). This cross‐linking was observed in both the apo form and the DNA‐bound state, and relative quantification showed only slight changes caused by DNA binding. This observation is consistent with available structures wherein contact between these regions is compatible with DNA binding to Ku70/80.

Overall, our mass spectrometric analysis corroborated the localization of SAP domain in the DNA aperture of the Ku70/80 complex as indicated by the EM density maps. Furthermore, mass spectrometric data provided additional information about fluctuating positions of the SAP domain and describe structural changes caused by DNA binding. The identified cross‐linking also showed an alternative position of the SAP domain compared with both our cryo‐EM data and the previous crystal structures.

### Structural modelling defined possible positions of the SAP domain

In order to integrate results from cryo‐EM and mass spectrometry, we performed structural modelling calculations. Specifically, we used a blind docking approach followed by evaluation with restraints derived from obtained cross‐links. Three docking solutions were obtained, which captured both the apo and DNA‐bound conformations.

**Fig. 3:**
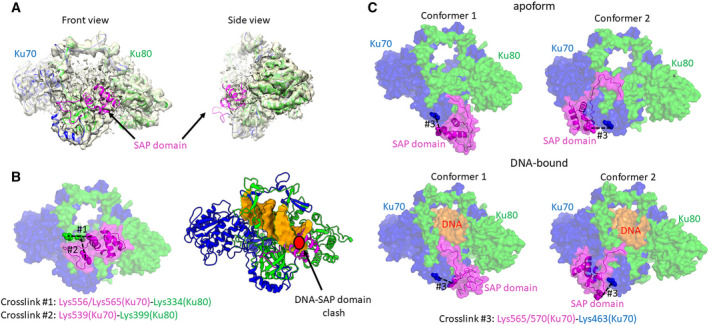
Structural modelling guided by cryo‐EM and mass spectrometry shows variable positions of the SAP domain. Parts of the Ku70/80 are coloured as follows: Ku70 subunit (aa 1–537), blue; the SAP domain (aa 538–609 in Ku70), magenta; Ku80 subunit, green; DNA molecule, orange. (A) The Ku70/80 apo form with the SAP domain localized in the DNA aperture as shown by structural model superposed with our cryo‐EM map. Structural images were generated by ucsf chimera. (B) The Ku70/80 apo form with the SAP domain in the DNA aperture as demonstrated by the cross‐links #1 and #2 in the apo form (left). This position of the SAP domain is not compatible with DNA binding as shown by superposition of the apo form with the DNA‐bound state (right). Structural images were generated by pymol (left) and chimerax (right). (C) The Ku70/80 apo form (upper part) and the Ku‐DNA complex (lower part) with the SAP domain localized under the DNA aperture in two possible positions as shown in conformers 1 and 2. These models are supported by the presence of cross‐link #3. Structural images were generated by pymol.

The first model of the Ku70/80 apo form was consistent with our cryo‐EM maps, such that two of the three cross‐links [cross‐links #1 – Lys 334 (Ku80) – Lys 556/565 (Ku70) and #2 – Lys 399 (Ku80) – Lys 539 (Ku70)] were satisfied (Fig. 3A,B). The occurrence of both these cross‐links between the SAP domain and the central domain of Ku80 was dramatically decreased in a DNA‐bound form of Ku70/80, supporting the suggestion that this mode of interaction requires the apo form and is disrupted by DNA binding (Fig. 3B). This model revealed C_α_‐C_α_ distances between Lys 334 (Ku80) and Lys 556 or Lys 565 (Ku70) of 10 and 20 Å, respectively, while the C_α_‐C_α_ distance between Lys 399 (Ku80) and Lys 539 (Ku70) was 19 Å.

The other two docking solutions obtained were consistent with the experimental evidence for both the apo and DNA‐bound complexes, with the interactions mediated by cross‐link #3 seen in both samples (Fig. 3C). In both solutions, the SAP domain was positioned distal to the DNA aperture. The C_α_‐C_α_ distances between Lys 334 (Ku80) and Lys 556 (Ku70) range from 24 to 65 Å, while the C_α_‐C_α_ distances between Lys 334 (Ku80) and Lys 565 (Ku70) range from 39 to 50 Å. The SAP domains in these models are closer to the helical arm under the DNA aperture. Particularly, the C_α_‐C_α_ distances between Lys 463 (Ku70) and Lys 565 (Ku70) range from 14 to 18 Å, while the C_α_‐C_α_ distances between Lys 463 (Ku70) and Lys 570 (Ku70) range from 12 and 16 Å.

In summary, structural modelling was consistent with experimental observations, with the SAP domain existing in multiple possible conformations in both the apo and the DNA‐bound forms.

## Discussion

The SAP domain is a 35‐residues long structural motif that is found in nuclear proteins involved in transcription, DNA repair or RNA processing [19]. It is directly implicated in multiple biological processes such as apoptotic chromatin condensation [20], DNA damage response [21] or splicing regulation [22]. Typically, the SAP domain simultaneously interacts with multiple binding partners and acts as a tether in multicomponent assemblies [23, 24]. The SAP domain is also located at the C‐terminal part of Ku70, a key component of the NHEJ pathway, but its structural arrangement and underlying biological function are still not well understood.

**Fig. 4:**
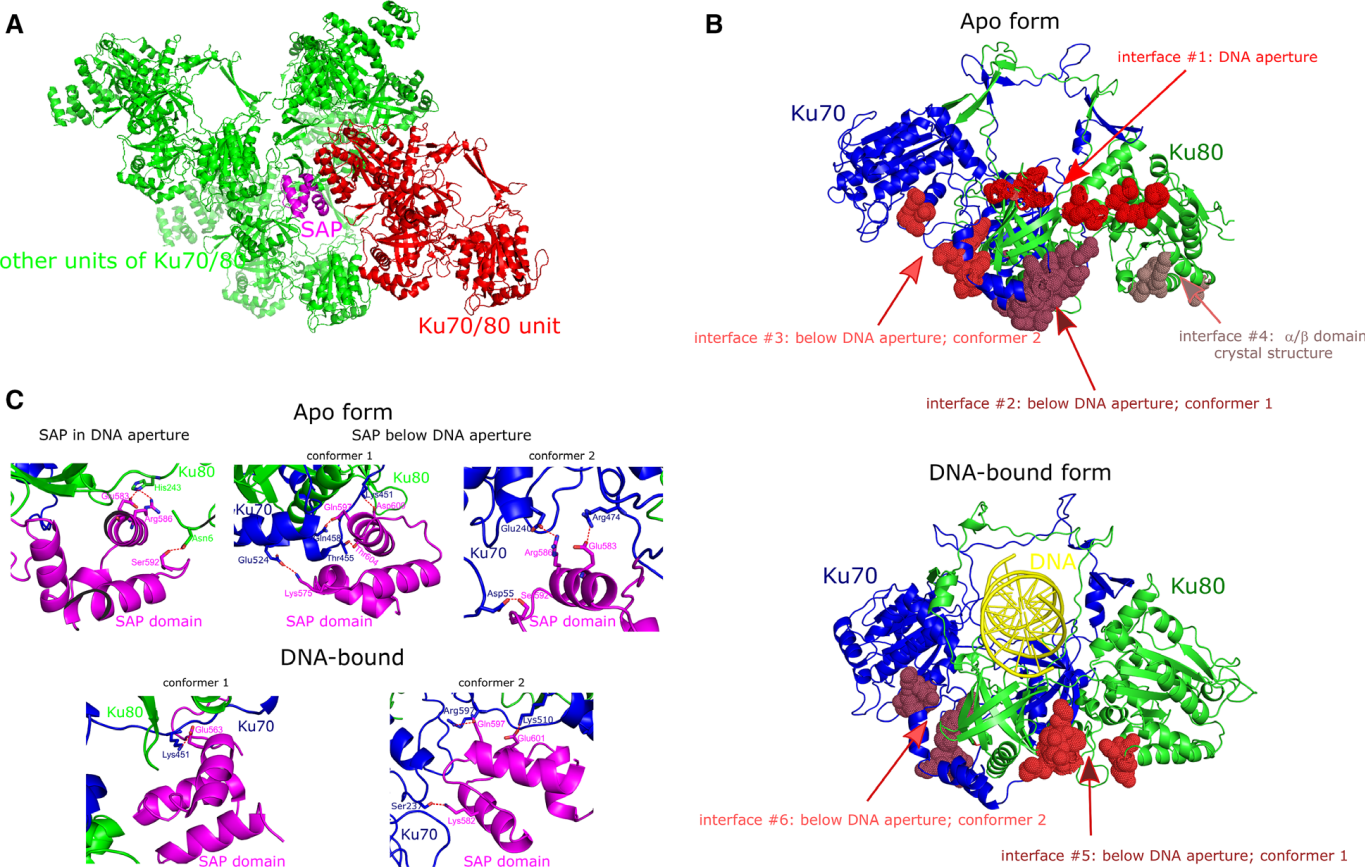
Interdomain interaction in the SAP domain. (A) Crystal packing in Ku70/80 apo form (PDB ID 1JEQ). The SAP domain is involved in multiple crystal contacts. One unit of Ku70/80 (red colour) is shown with SAP domain (magenta) which is packed against surrounding Ku70/80 units (green). (B) Potential interaction areas for the SAP domain in Ku70/80 revealed by our integrative modelling (interfaces 1–3 and 5–6 in apo and DNA‐bound form, respectively) and by previous crystal structure (interface 4; PDB ID 1JEQ). Interfaces are highlighted as dots in varying shades of red colour. (C) Detailed interactions of the SAP domain. Potential positions of the SAP domain were defined by integrative modelling guided by cryo‐EM map and mass spectrometry. Ku70/80 apo form (upper part) and Ku‐DNA complex (lower part) with the SAP domain localized either in the DNA aperture or under the DNA aperture in two possible positions as shown in conformers 1 and 2. Each part of the Ku70/80 is coloured as follows: Ku70 subunit (aa 1–537), blue; the SAP domain (aa 538–609), magenta; Ku80 subunit, green; DNA molecule, orange. Hydrogen bond and ionic interactions are highlighted as dash red lines. Structural images were generated by pymol.

Using intermolecular cross‐linking coupled with cryo‐EM and mass spectrometry, we captured the fluctuating position of the SAP domain in Ku70/80. Demonstrating its high positional flexibility, the SAP domain was found in three positions. In the first position, the SAP domain is localized in the DNA aperture. This position was observed only in the apo form as DNA binding caused a displacement of the SAP domain. The second and third positions were below the DNA aperture close to the helical arm of Ku70 (aa 450–538), which is fully compatible with a structure for Ku‐DNA complex. All these regions differ from the position of the SAP domain described in the earlier crystal structure [5]. Differences may be attributed to changes induced by crystal packing or varying experimental conditions for each technique that enabled capturing only a subset of positions for the SAP domain in the Ku70/80 complex. Notably, the SAP domain is involved in multiple crystal contacts in Ku70/80 apo form (PDB ID 1JEQ; Fig. 4A). Combining our present study and previously described crystal structure [5], we define an area for the movements of the SAP domain in the apo form and DNA‐bound states of Ku70/80 (Fig. 4B).

The NMR structure of the SAP domain [6] revealed a conserved positive patch on the surface formed by Lys582, Arg586, Lys591, Lys595 and Lys596. Based on the changes in chemical shift, Zhang *et al*. hypothesized that residues of this positive patch might be directly involved in DNA binding. In particular, Lys582 and Arg586 may interact with the major groove of DNA, while Lys591, Lys595 and Lys596 may interact with phosphate groups of the DNA backbone. However, in our models of the DNA‐bound Ku70/80, none of these residues interacts with the DNA as they are separated by at least 25 Å. One may speculate that the SAP domain may form transient interactions with DNA which is specifically formed during the initial recognition of DNA breaks before getting displaced from the DNA aperture.

Regarding the residues of the positively charged patch on the SAP domain, we observed their engagement only in isolated interdomain interactions within the Ku70/80 in our models (Fig. 4C). This further supports the notion about the transient nature of the interactions between the Ku70/80 core and the SAP domain.

In order to capture snapshots of the flexible arrangements in the Ku70/80 complex, we utilized chemical cross‐linking followed by structural characterization. Indeed, cross‐linking has been previously applied for studying multiple dynamic protein‐DNA assemblies, such as the MutH‐MutL complex in DNA mismatch repair [25], the monoubiquitin ligase in Fanconi anaemia pathway [26] or protein–nucleosome complexes [27].

**Fig. 5:**
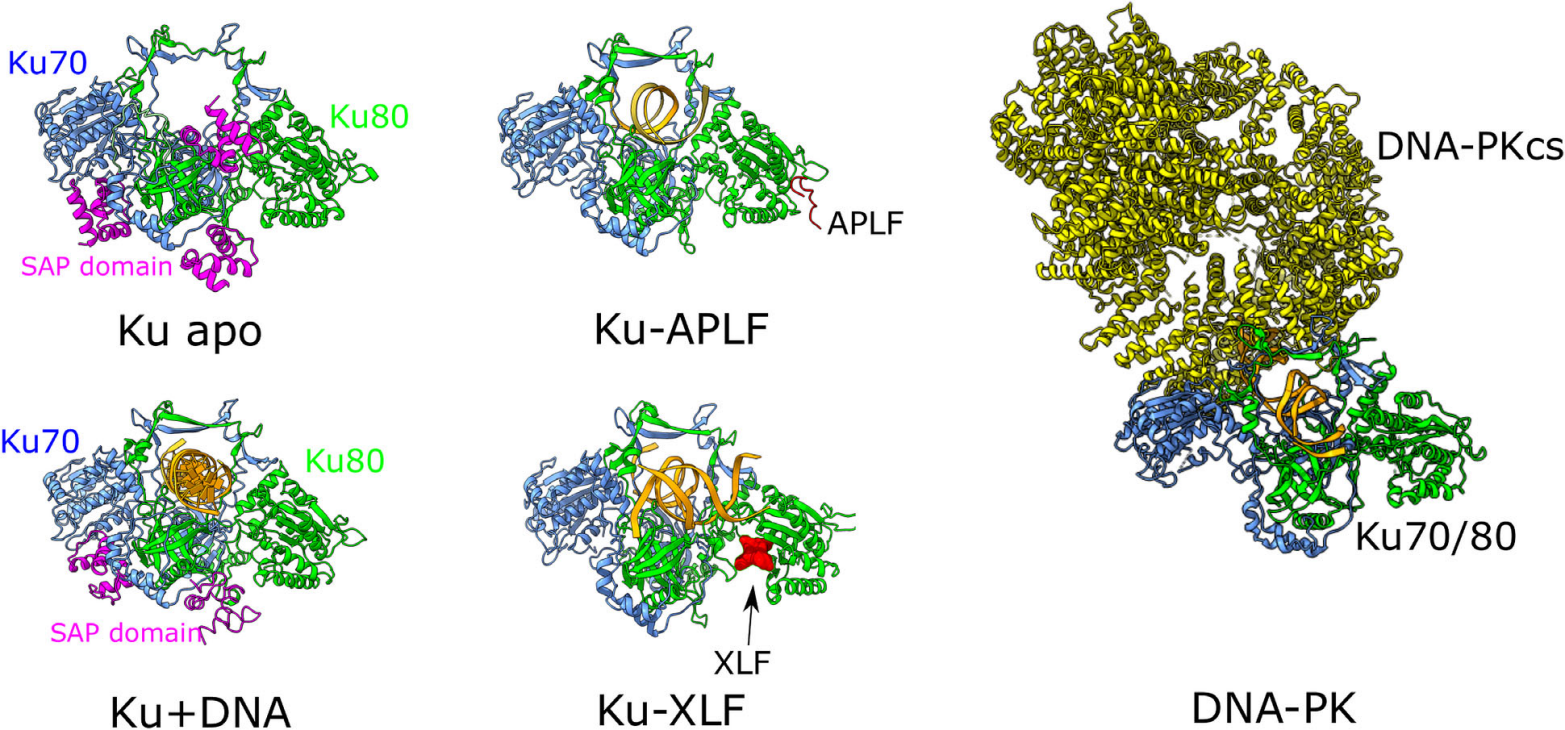
Distinct positions of the SAP domain and Ku‐binding partners in our structural models and the available experimental structures. Illustration of Ku70/80 apo form and Ku‐DNA complexes shows positions of the SAP domain (highlighted as magenta cartoon) in the Ku70/80 complex. Experimental structures for Ku‐APLF (PDB ID 6ERF), Ku‐XLF (PDB ID 6ERH) and DNA‐PK (PDB ID 6ZHA) show binding sites for the interacting proteins. Structural images were generated by chimerax.

Our study is the first report of localization of the SAP domain in the DNA aperture of Ku70/80. Comparison with described structures for Ku‐APLF and Ku‐XLF [28] and DNA‐PK [29, 30] showed that other binding partners from the NHEJ pathway do not interfere with the defined position for the SAP domain (Fig. 5). Our findings suggest that the SAP domain could be a part of the entry gate for the initial recognition of DNA breaks [31]. Interestingly, the SAP domain has been reported to bind both the DNA [6] and TFs from the homeobox family [13], suggesting a role in transcription. Furthermore, several studies demonstrated that NHEJ mainly acts on transcribed genes and that interaction of NHEJ components with the transcription machinery is essential for efficient DNA repair [32, 33]. The SAP domain may thus represent an important structural motif linking NHEJ and transcription. For instance, it might recognize DNA breaks in transcriptionally active genes through interaction with TFs, which are subsequently removed from DNA thus enabling efficient repair via NHEJ. Notably, the SAP domain in SAF‐B can tether proteins from the pre‐mRNA processing pathway with transcriptionally active chromatin [24]. In conclusion, this work uncovers structural dynamics of the SAP domain in Ku70 and provides essential information for elucidating its role in NHEJ and other biological processes.

## Materials and methods

### Protein preparation

Ku70/80 heterodimer was expressed in Sf9 cells and purified as described previously [34]. Briefly, proteins were purified using immobilized metal affinity chromatography followed by ion exchange chromatography (Mono‐Q column; GE Healthcare, Chalfont St. Giles, UK) and gel filtration (Superdex 200; GE Healthcare) according to published procedures [34]. The proper function of the purified proteins was checked using electrophoretic mobility assay with 6‐FAM labelled 20 bp oligonucleotide (CTGATGCGTCGTCGGACTGA). In this study, both the full‐length protein and the truncated variant lacking Ku80 CTD (Δ 566–732 as in [5], where the heterodimer is known as Ku70/tr80) have been used. For structural studies, we used Y‐shaped oligonucleotide composed of the following strands: CGCGCCCAGCTTTCCCAGCTAATAAACTAAAAACTATTA and TAATAGTTTTTAGTTTATTGGGCGCG. The oligonucleotide was mixed with Ku70/80 in an equimolar ratio and preincubated at room temperature for 10 min.

### Cryo‐electron microscopy

#### Sample preparation

Ku70/80 proteins (0.4 mg·mL^−1^) in 20 mm sodium phosphate (pH 7.0) containing 50 mm potassium chloride and 1 mm DTT (cross‐linking buffer) were subjected to intermolecular cross‐linking using DSA (Creative Molecules Inc.) with protein‐cross‐linker molar ratios of 1–20 and 1–50. After the cross‐linking reaction (at room temperature for 1 h), proteins were fractionated using gel filtration (Superose 6/3.2; GE Healthcare). Gel filtration was also used for the fractionation of unmodified proteins. Fractions containing Ku70/80 were collected, dissolved in cross‐linking buffer containing 0.05% octyl glucopyranoside (Avanti Polar Lipids, Alabaster, AL, USA), applied to Quantifoil (Cu R1.2/1.3, 300 mesh) holey carbon grids (Agar Scientific, Stansted, UK) and vitrified in liquid ethane.

#### Data acquisition

Cryo‐EM data sets were collected in the Department of Biochemistry, University of Cambridge (Ku70/80 bound to DNA and uncross‐linked Ku70/80 apo form) or the Electron Bio‐Imaging Centre, Diamond Light Source (cross‐linked Ku70/80 apo form). Cryo‐EM data were collected on a Titan Krios electron microscope (Thermo Fisher Scientific, Eindhoven, The Netherlands) operated at 300 kV using a K2 or K3 detector (Gatan Inc., Pleasanton, CA, USA) in a counting mode. Data were recorded under conditions of ~ 49.5 and 39.3 e‐/Å^2^ for 40 frames (Ku‐DNA and cross‐linked apo form), or 53.5 e‐/Å^2^ for 48 frames (uncross‐linked apo form) and a defocus range of −1.2 to −2.7 µm (−0.9 to −2.7 to µm for Ku‐DNA complex). Magnification setting resulted in a pixel size of 1.07 Å (Ku‐DNA), 1.048 Å (cross‐linked apo form) and 0.325 Å (uncross‐linked apo form).

#### Data processing

For the Ku‐DNA complex and the cross‐linked apo form, MotionCor2 [35] and Gctf [36] were used for motion correction and CTF estimation. Particles of the cross‐linked Ku70/tr80 apo form were picked using CrYolo [37]. Data were processed in Relion 3.1 using standard procedures [38]. In the case of the uncross‐linked apo form, motion correction, CTF estimation and particle picking were performed using Warp [39] and processing was done using CryoSparc [40].

After initial 2‐D classification, particles were sorted through 3D classification. Typically, 25–50% of the particles were included in the higher resolution 3‐D class for the Ku70/80 heterodimer. Other 3‐D classes contained dissociated dimer or protein complex of poor resolution. The particles from the selected 3‐D class were further filtered and refined. An overall resolution of the final maps was calculated by the Fourier shell correlation at 0.143 cut‐off (Fig. 1B).

#### Model refinement

Available structural models (PDB ID 1JEY for the Ku‐DNA complex and PDB ID 1JEQ for the Ku apo form) were docked into resulting maps using phenix (version 1.16‐3546, Phenix, Berkeley, CA, USA). Structural models were rebuilt according to the EM map in coot (version 0.892, Coot, MRC MLB, Cambridge, UK) and subsequently refined using real‐space refinement in phenix. Resulting models were visualized in pymol Viewer 1.5 (Schrodinger Inc., New York, NY, USA), ucsf chimera (version 1.13.1, UCSF Chimera, San Francisco, CA, USA) and chimerax (version 1.1, ChimeraX, San Francisco, CA, USA) [41]. Details of Cryo‐EM data collection, model refinement and validation are shown in Table 2.

**Table 2:** Cryo‐EM data collection, refinement and validation statistics.

	Ku70/80 apo (EMDB ID_1292111939) (PDB 7AXZ)
Data collection and processing	
Magnification	130 000
Voltage (kV)	300
Electron exposure (e–/Å^2^)	39.3
Defocus range (μm)	−1.20 to −2.7
Pixel size (Å)	1.048
Symmetry imposed	/
Initial particle images (no.)	568 771
Final particle images (no.)	226 000
Map resolution (Å)	3.2
FSC threshold	0.143
Refinement	
Initial model used (PDB code)	1JEQ
Map sharpening *B* factor (Å^2^)	−40
Model composition	
Nonhydrogen atoms	16 566
Protein residues	1023
*B* factors (Å^2^)	
Protein (min/max/mean)	36.53/152.56/89.30
Rmsd	
Bond lengths (Å)	0.009
Bond angles (°)	0.873
Validation	
MolProbity score	2.13
Clash score	11.35
Poor rotamers (%)	0.00
Ramachandran plot	
Favoured (%)	89.81
Allowed (%)	10.19
Disallowed (%)	0.00

### Protein cross‐linking and mass spectrometry

Proteins (0.4 mg·mL^−1^) in 20 mm sodium phosphate (pH 7.0) containing 50 mm potassium chloride and 1 mm DTT were treated with an equimolar mixture of DSA^12^C_6_ and DSA^13^C_6_ cross‐linkers in protein–cross‐linker ratios of 1–20 and 1–50 for 1 h at room temperature. Proteins were digested in a solution using trypsin (Promega Corporation, Madison, WI, USA) for 8 h at 37 °C and subjected to analysis using liquid chromatography coupled with Fourier transform ion cyclotron resonance mass spectrometry (LC‐FT ICR MS) as described previously [42].

### Structural modelling

The interactions of the SAP domain with Ku70 were modelled using Schrodinger (2018‐4). The structure of the SAP domain (aa 559–609) was modelled using the experimental NMR structure (PDB ID: 1JJR, [6]). We applied a blind docking approach, with the top solutions evaluated by their ability to satisfy the experimental cross‐linking restraints. For the poses where the SAP domain was located near empty electron density at the dimerization interface, we applied a constraint from 6 to 18 Å on the distance between S244 and T577 of Ku70. For the models where the SAP domain was not within this electron density, two constraints from 10 to 14 Å on the distances between K463 and K565/K570 of Ku70 were applied. The flexible loop from residue G538 to E558 was subsequently added to the three poses satisfying these restraints using Modeller version 9.24 [43]. The loop was built using the automodel command and refined using slow mode.

## Conflict of interest

The authors declare no conflict of interest.

## Author contributions

AH conceived the study, performed experiments and wrote manuscript; PT, LK, RB and AC analysed EM data; ZK and PN analysed MS data; TBN and DBA performed structural modelling; TLB conceived the study. All authors revised manuscript.

### Peer Review

The peer review history for this article is available at https://publons.com/publon/10.1111/febs.15732.
